# Retraction Note: Exosome-mediated transfer of lncRNA PART1 induces gefitinib resistance in esophageal squamous cell carcinoma via functioning as a competing endogenous RNA

**DOI:** 10.1186/s13046-023-02648-7

**Published:** 2023-04-06

**Authors:** Min Kang, Meiping Ren, Yan Li, Yuqiong Fu, Minmin Deng, Changping Li

**Affiliations:** 1 Department of Digestive Diseases, Affiliated Hospital of Southwest Medical University, Luzhou, Sichuan China; 2 Drug Discivery Research Center, Southwest Medical University, Luzhou, Sichuan China; 3 Molecular Medicine Experimental Center, Affiliated Hospital of Southwest Medical University, Luzhou, Sichuan China; 4 Department of Respiratory Medicine, Affiliated Hospital of Southwest Medical University, Luzhou, Sichuan China

**Retraction Note:** ***J Exp Clin Cancer Res*** **37, 171 (2018)**

10.1186/s13046-018-0845-9

The Editor-in-Chief has retracted this article. Concerns were raised regarding a number of figures, including but not limited to:

- Figure 1a appears to be identical to Figure 2B in an article by different authors describing different studies that was simultaneously under consideration at a different journal [1] but flipped horizontally.
- Figure 2f appears to overlap with Figure 2F in a previously published article [3] and Figure 4C in an article by different authors that was simultaneously under consideration [4].
- Figure 3e appears to be identical to Figure 2c in a previously published article [2].
- Figure 7c appears to be identical to Figure 5B in an article by different authors describing different studies that was simultaneously under consideration at a different journal [1].
- Figure 8a appears to be identical to Figure 6A in an article by different authors describing different studies that was simultaneously under consideration at a different journal [1].

The Editor-in-Chief therefore no longer have confidence in the results and conclusions of this article.

The authors have not responded to correspondence regarding this retraction.
